# The oral administration of bacterial extracts prevents asthma via the recruitment of regulatory T cells to the airways

**DOI:** 10.1186/1479-5876-9-S2-P22

**Published:** 2011-11-23

**Authors:** Nicolas Glaichenhaus, Valérie Julia

**Affiliations:** 1 Université de Nice-Sophia Antipolis, Institut National de la Santé et de la Recherche Médicale, Valbonne, France; 2 Université de Lille 2, Institut National de la Santé et de la Recherche Médicale, Lille, France

## Background

The prevalence of asthma has steadily increased during the last decade, probably as the result of changes in the environment, including reduced microbial exposure during infancy. Accordingly, experimental studies have shown that deliberate infections with live pathogens prevent the development of allergic airway diseases in mice. Bacterial extracts are currently used in children suffering from repeated upper respiratory tract infections. In this study, we have investigated whether bacterial extracts, commercially available as Broncho-Vaxom (BV), could prevent allergic airway disease in mice.

## Results

Oral treatment with BV suppressed airway inflammation through IL-10-dependent and MyD88-dependent mechanisms and induced the conversion of FoxP3-negative T cells into FoxP3-positive regulatory T cells. Furthermore, CD4-positive T cells purified from the trachea of BV-treated mice conferred protection against airway inflammation when adoptively transferred into sensitized mice.

## Conclusion

Treatment with BV could possibly be a safe and efficient strategy to prevent the development of allergic disease in children.

